# Is there a Profile of Spontaneous Seizure-Alert Pet Dogs? A Survey of French People with Epilepsy

**DOI:** 10.3390/ani10020254

**Published:** 2020-02-05

**Authors:** Amélie Catala, Patrick Latour, Hugo Cousillas, Martine Hausberger, Marine Grandgeorge

**Affiliations:** 1 CNRS, EthoS (Éthologie animale et humaine)-UMR 6552, Univ Rennes, Normandie Univ, F-35000 Rennes, France; hugo.cousillas@univ-rennes1.fr (H.C.); martine.hausberger@univ-rennes1.fr (M.H.); marine.grandgeorge@univ-rennes1.fr (M.G.); 2 Association Handi’Chiens, 13 Rue de l’Abbé Groult, 75015 Paris, France; 3 Etablissement Médical de la Teppe, 25 Avenue de la Bouterne, 26600 Tain-l’Hermitage, France; patrick.latour@teppe.org

**Keywords:** seizure-alert dog, service-dog, epilepsy, seizure detection, human–dog relationship, dog personality

## Abstract

**Simple Summary:**

Very little is known about dogs that could alert their owner of an impending epileptic seizure. Here, we explored the profiles of untrained dogs that spontaneously show seizure-related behaviors. Using a self-reporting questionnaire, we found that these dogs do not have a particular profile (e.g., sex, breed, age, epilepsy of the owner), but bonding is perceived as better when the dog alerts compared to a dog who does not alert. Personality traits helped discriminate between these two types of dogs; seizure-alert dogs were scored higher for Motivation, Training Focus and lower in Neuroticism than non-alerting dogs. In addition, we reported alert-related behavior characteristics (e.g., the dogs that alerted the more frequently stayed close and stared at their owner when he/she had a seizure). Our results are in line with the existing literature and help further the understanding of seizure-alert dogs. In particular, we suggest that some personality traits could be a basis for the selection of future assistance dogs.

**Abstract:**

Despite controversies and the lack of research, dogs are empirically selected and trained to perform as service dogs, in relation to the dogs’ and future owners’ characteristics. We assessed the characteristics of both humans and dogs in an unbiased population (not selected or trained) of spontaneous seizure-alert by pet dogs and investigated whether we could replicate previous findings. We addressed a self-reporting questionnaire to French people with epilepsy. We analyzed the general characteristics of the humans and pet dogs and their behaviors that could alert their owner before a seizure. In addition, we used the Monash Canine Personality Questionnaire refined to evaluate pet dogs’ personality through five different traits, and the Monash Dog-Owner Relationship scale to assess human–dog relationships. In line with previous reports, we found no particular factor, either pet-, people- or epilepsy-related that could be associated with the presence or absence of alert behaviors. Alert behaviors and circumstances were explored and three different alert patterns emerged. In terms of personality, seizure-alert pet dogs scored significantly higher than non-alerting dogs for the traits “Motivation” and “Training Focus” and lower for “Neuroticism”. The owner–dog bond score was significantly higher for seizure-alert dogs than for non-alerting dogs.

## 1. Introduction

Recognition of the value of animal companionship for human health is growing [1,2]. The numbers and roles of service dogs are expanding globally [3]. According to US legislation, a “service dog” is a dog trained individually to perform tasks for the benefit of an individual with a disability [4]. Many roles for dogs have emerged, such as guides for blind people, mobility assistance, or detection of hypoglycemia episodes (e.g., Hardin, Anderson, and Cattet [5]; Rooney, Morant, and Guest [6]).

More recently, there has been an interest in service dogs that could help people with epilepsy called seizure dogs [7]. These dogs can have two roles: to alert their owner of an impending seizure (i.e., seizure-alert dogs, SAD), and/or to demonstrate specific behaviors during or immediately after a seizure (i.e., seizure-response dogs, SRD). Although charity organizations specifically train dogs for those purposes, pet dogs (i.e., without specific training) have been reported to present such behaviors. However, at present, there has been no direct scientific observation of a dog’s behavior alerting an epileptic seizure [7,8]. It is also unknown what the dogs could perceive during a pre-ictal period [9,10,11]. In this context, most trainers select dogs that show an increased interest toward humans and are motivated by play or food, facilitating training as a service dog. Other organizations select future service dogs on the basis of their temperament, choosing in particular dogs that have a natural tendency to be anxious, as it is believed that anxiety makes them “worry” about their handler having a seizure [12].

In fact, there are two issues concerning dogs for people with epilepsy: their ability to perceive something, and their facility to indicate their discrimination non-ambiguously. In this respect, the relationship established between a dog and its owner is a potentially important issue. Moreover, recently, we (Catala et al. 2019) showed that trained dogs can discriminate a “seizure odor” from the same person’s body odors sampled in other contexts. This demonstrates that there is a potential for dogs to use olfactory cues for detection. However, it is still unknown whether olfactory cues are the sole sensory ability used.

Since alert displays may also be performed by dogs outside the time frame of a seizure and also by untrained pet dogs, a major issue is whether all dogs are able to discriminate seizure odors reliably. It is important to evaluate dog traits that might potentially be important to convey efficiently that they have discriminated.

Pet dogs that had acquired alert and/or response behavior spontaneously are especially interesting in this respect. Four studies reported characteristics of such pet dogs through questionnaire studies. The earliest study [13] reported that out of 29 subjects who owned dogs, 9 said that their dogs responded to a seizure, and three of these nine dogs were reported to exhibit alert behaviors. These pet dogs demonstrated “attention-getting” behaviors towards their owners approximately three minutes before the start of a seizure [11] (10 s to 5 h): they whined, paced in front of or around the person, barked or stared at the person. The authors did not find any age, breed, sex or size effect on the propensity to perform SAD/SRD behaviors. Kirton [11] found similar proportions of alert and response pet dogs in a study of 48 families; they found that more larger (>18 kg) and female (80%) dogs presented SAD behaviors, representing a large variety of breeds (n = 8). There was a median sensitivity of 80% (i.e., proportion of a seizure being alerted) and no false alert. Alert occurred during the first seizure in 60% of the families and developed within a month for the rest. In this study, the reported alert behaviors were, amongst others: sitting on the person and preventing her/him to stand up, pushing the person away from stairs and licking his/her feet. These observations converge with those reported by Pinikahana and Dono [14] on the basis of 12 respondents’ answers: the pet dog would mainly sit or stand close to and observe the person (48.6%) and/or sit or lean on the person (35.1%). These three studies [11,13,14] relied upon questionnaires to explore the behavior of seizure-alert pet dogs as a quick way to assess various factors of influence on this uncommon event. More recently, another questionnaire [15] was developed and used in six countries (Belgium, Germany, Italy, UK, US, Spain) with the aim of assessing the behaviors displayed by seizure-alerting dogs (trained and untrained) and to identify patients-or dog-related factors of influence on the presence or absence of these behaviors. A total of 132 of the 277 people who answered said they owned a spontaneous seizure-alerting dog, and 10 owned a trained seizure-alert dog. Two validated scales were used in addition to the general questionnaire: the Monash Dog–Owner Relationship [16] and the Monash Canine Personality Questionnaire-Revised (MCPQ-R) [17]. The results showed that except for the presence of preictal symptoms, no factor associated with the presence of spontaneous alerting behavior could be evidenced, but they noted that the owner–dog bond was significantly higher for SAD than for non-alerting dogs (NAD). Moreover, scores for SAD were significantly higher in the personality traits “Amicability”, “Motivation”, and “Training focus” than for NAD. However, people with preictal symptoms were possibly over-represented, mostly probably with ictal symptoms (i.e., aura; defined as a focal seizure involving subjective sensory or psychic phenomena only [18]) as 68% of these symptoms occurred between less than 1 min to 5 min before a seizure.

The aim of the present study was to go further by identifying dog traits that could favor this type of discrimination and their ability to indicate it to owners. We used a modified version of the Martos-Martinez et al.’s [15] questionnaire to replicate this study, but on a culturally different population, and expanded the questions of alert behaviors, behavioral profiles and circumstances in which the behaviors could occur.

## 2. Materials and Methods

### 2.1. Ethics Statement

The survey complies with the French law on digital information and has been registered by CNIL registered data protection officer (Correspondant Informatique et Libertés CIL) of the University of Rennes 1 (CIL–N 2017-DN-005). Survey participants were fully informed about the purpose and background of the study. As the study survey was entirely anonymous, informed signed consent was not required from participants. Participants could abandon the online survey anytime.

### 2.2. Subjects

The survey was available online from June 2017 to August 2018 on Limesurvey. Epileptic people were recruited on a voluntary basis, throughout several networks: a medical center on epilepsy (Etablissement Medical de La Teppe, Tain l’Hermitage, France), associations of patients with epilepsy (e.g., Epilepsie France, Association Paratonnerre) and a French association of service dogs (Handi’Chiens). They received a request and instructions in an email with an access to the anonymous online survey. During the subjects’ enrolment period, a total of 228 people participated. In this study, we define a service dog as a dog specifically trained for a person with epilepsy. In these terms, no service dog owners participated, but some owners of dogs trained for other purposes (e.g., mobility assistance) were included as their dog spontaneously developed epilepsy-specific behaviors.

### 2.3. Questionnaire Design

We aimed to collect information from people with epilepsy living with a dog. In order to collect information, the questionnaire used was a modified version of Martos Martinez-Caja et al.’s [15]. Criteria included were having been diagnosed with epilepsy (although it was a self-reported diagnosis) and currently or frequently living with a dog. Owning a dog displaying alerting behavior was not a requirement to participate, as we were also interested in examining differences between owners of dogs with and without alerting behaviors.

The online survey consisted of seven sections, distributed in a General questionnaire part and the two validated questionnaires (MPCQ-R and MDORS) (cf. suppl. Mat.).

### 2.4. General Questionnaire

A statement on the pet dog’s behavior related to a seizure (i.e., alert, response, both alert and response, no specific behavior toward seizure).

Characteristics of seizure-alerting behaviors (e.g., «bark», «stare») throughout 22 items based on anticipatory behaviors frequently found in literature [11,13,14,15] and an open answer “other”.

Pet dog characteristics (i.e., sex, neuter status, age, size and breed) and characteristics of pet-ownership (e.g., time spent close, involvement of the person in daily tasks such as feeding or walking).

Owner’s epilepsy characteristics (e.g., date of diagnosis, epilepsy and seizure type notably if there is an aura, seizure frequency, auras).

Owner demographics (e.g., sex, date of birth, regional location, country). 

Monash Canine Personality Questionnaire-Revised (MPCQ-R) [17] was used to assess dog personality traits. It is a questionnaire that measures canine personality along five dimensions: Extraversion, Motivation, Training Focus, Amicability and Neuroticism. Each item of pet dog personality was scored from 1 (“really doesn’t describe my pet dog”) to 5 (“really describes my pet dog”).

Monash Dog–Owner Relationship Scale (MDORS), a validated questionnaire developed by Dwyer et al. [16] that aims at describing human–dog relationships. Questions contribute to three subscales, (1) Dog–Owner Interaction (DOI), (2) Perceived Emotional Closeness (PEC), and (3) Perceived Costs (PC). The answers to subscales 1 and 2 were labeled 1–5 (very seldom/totally disagree–very often/strongly agree), and the answers to subscale 3 were labeled 5–1 (very seldom/disagree–very often/strongly agree). 

MPCQ-R and MDORS were back-translated to French. Data were collected throughout close-ended questions (e.g., yes/no, male/female), open-ended question (give precision with “other” items) and 5-points-Likert scale such as for dog personality traits with 1 being “really doesn’t describe my dog” and 5 being “really describes my dog”. As in Martos Martinez-Caja et al.’s [15], our survey included both MCPQ-R and MDORS, though with a 5-points-Likert scale instead of a 6-points-Likert scale due to technical limitations. Themes addressed in sections of the General questionnaire are comparable with the ones addressed in Martos Martinez-Caja et al.’s [15], although the questions are not exactly the same.

### 2.5. Data and Statistical Analysis

Only questionnaires with at least 5 of the 7 sections completed were kept for further analysis. Thus, 72 people were included in this study (i.e., exclusion of 156 questionnaires not sufficiently completed). All were French natives, lived with epilepsy and nine parents helped their child to complete their questionnaire. All participants owned at least one pet dog, and two owned more than one dog (i.e., two questionnaires were completed, one per pet dog as we considered here the dog as the statistical unit).

χ^2^ one-sample tests [19] were used to assess whether each parameter (e.g., sex of the owner, sex of the dog, seizure frequency) differed significantly between the seizure alert group and the general population.

In the literature, some studies used the sum of the scores for each item [20] while others used their mean [21]. In the absence of a defined procedure, the MCPQ-R and MDORS scores were calculated by summing the scores of each item constituting a personality trait (MCPQ-R), or a subscale (MDORS), as well as by calculating the mean scores of these items. The overall score was also calculated for the MDORS. As SAD and NAD were two independent populations, scores for the five traits of personalities of SAD and NAD were compared using Mann–Whitney tests. These tests were also used to compare the MDORS scores of SAD and NAD, for total scores as well as for the three different subscales: Dog–Owner Interaction, Perceived Emotional Closeness, and Perceived Costs.

In addition, in order to determine a behavioral profile of a spontaneously seizure-alerting dog, Kruskall–Wallis tests were performed on seizure-alert dogs’ personality traits.

As some data were qualitative, we performed a multiple correspondence analysis (MCA) on alert behaviors followed by a hierarchical cluster analysis (HCA). Ward’s method was applied on the results of the MCA [22], and a dendrogram plot was generated, where the vertical axis set indicated the loss in within-cluster similarity (the variance increase) as clusters merged. This was used to select the cluster solution for the later analysis and a three-cluster solution was chosen as it presented the greatest between-cluster distance. The resulting model was explored using Fisher-exact tests to identify the owner and pet dog characteristics, which contributed to group membership. Each profile was represented by a radar plot for anticipatory behavior traits, and each peak of the plot represented a single trait (approach similar to Grandgeorge et al.’s [23]).

We used XLSTAT software to perform these analyses and significance values of *p* < 0.05 were considered significant for the purposes of this study. 

## 3. Results

### 3.1. Information about the Owners

We had a total of 72 participants (38.6% men and 61.4% women, average age: 29.2 ± 13.5 years old, range: 6 to 66 years) (Table 1).

All types of seizures were present in the respondent population, but they were not equally distributed: 75% had seizures of generalized onset, 6.3% focal aware seizures and 6.3% focal seizures with impaired awareness. A total of 12.5% reported experiencing more than one type of seizure on the basis of the onset, i.e., seizures from both focal and generalized (i.e., focal to bilateral tonic-clonic seizure or generalized onset) (6.3%), or generalized and psychogenic nonepileptic (6.3%) seizure. Characteristics of epilepsy were presented in Table 2.

### 3.2. Information about the Dogs with Anticipatory Behaviors

Twenty-two (30.6%) of the 72 dogs were reported by owners to demonstrate seizure-alert behaviors. The dogs included in our survey as seizure-alert dogs were mostly purebred (73.7% purebred, 26.3% crossbred dogs) with a balanced sex ratio (55% males, 45% females). More than ten breeds were represented: Golden Retriever, Labrador Retriever, English Cocker Spaniel, Cavalier King Charles Spaniel, Boxer, Chihuahua, Yorkshire Terrier, Beauceron, Border Collie, Australian Shepherd, German Shepherd, Bernese Mountain Dog. Characteristics of the dogs are presented in Table 3.

**Figure 1:**
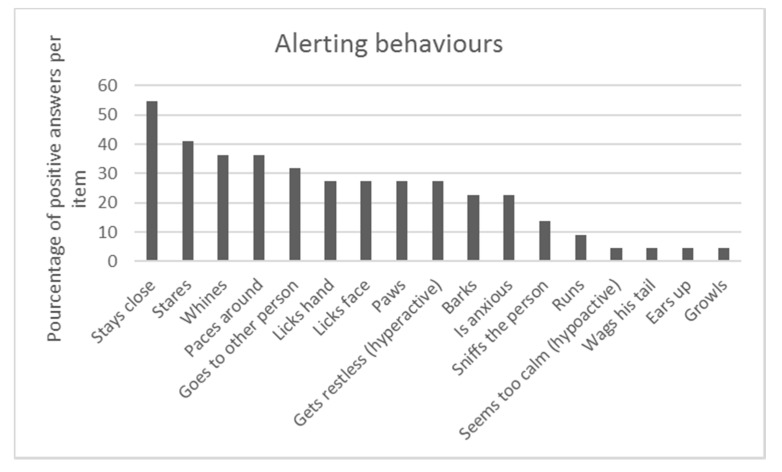
Dogs’ alerting behaviors (in percent).

Seizure alerting behaviors were diverse, see Figure 1. Behaviors that were reported the most commonly were staying very close to the person (54.5%), staring (40.9%), whining (36.4%), pacing around (36.4%), going to another person (31.8%) and licking their face or hand (27.3%). Three participants (13.6%) reported, in the category “other behavior”, that their dogs could “gently bite” (bite without pressure) to alert. Pet dogs were described mostly as being but not systematically in the same room (47.6%) or always (38.1%) in the same room. When the alert behavior was displayed, a majority of owners reported that their pet dog was looking at them (60%), although the majority were not in contact with their pet dog the moment before the alert (59.1%). The alert behavior often developed from the first day the pet dog was exposed to a seizure (45.5%), within the first week for 18.2%, one month for 13.6% or after a few months for 22.7%. Almost half of the owners reported that their pet dogs demonstrated seizure-alert behaviors for each seizure (43.8%), but a third reported that this was not the case (37.5%), while 18.7% did not know.

Circumstances when seizure alert behaviors were reduced, or not present, were “distance”, “night”, “opaque” physical barrier (e.g., wall) (all 18.2%) followed by “sleep of pet dog” (13.6%), “transparent physical barrier (e.g., French door)” (9.1%). “Obscurity” and “presence of strong scents” were minor (4.6%) while “presence of noise” was not reported.

Anticipation time varied among pet dogs, ranging from less than a minute (6.7%) to hours before (6.7%). Nevertheless, a majority of owners reported intervals of 5 to 10 min before a seizure (46.7%). This timing was not always the same before each seizure for 55.6% of the pet dogs, but it was the case for the remaining 44.4%. Most of the owners reported using an alert to put themselves in security (sit or lie down, 68.2%) and a third (31.8%) to warn their relatives or caretakers. In addition, a minor proportion of participants reported that they took medication after their pet dog’s alert (18.2%).

Some of the participants gave examples of seizure alert behaviors, such as “at the beginning, I was playing in the garden with my friend and, suddenly, the dog came and he was staring at me, went around me. We entered the house, then I lied down (seizure) and he stayed in front of me, nobody could approach, he guarded me.” Another testimony was that “my dog alerts my wife and sticks to me. She brings back toys. She does a lot of hugs and lickings. Then she lets my wife manage but stays nearby.”

### 3.3. Alerting vs. Non-Alerting Dogs

The only factor that differentiated seizure-alert and the general population of pet dogs was the number of hours spent by the owner with the pet dog per day: owners who spent the longest time daily with their pet dogs were more numerous to report that their dogs were seizure-alert pet dogs (between 18 to 24 h together; 62.5% for seizure-alert dogs versus 59.4% for general population; χ^2^ = 18.04, *p* < 0.001).

We could not find evidence for any other differences between seizure-alert and the general population of pet dogs for any other parameter (Table 3). All chi-square tests were non-significant (*p* > 0.7) for criteria relative to the dog (Table 3) as for the epilepsy related criteria (*p* > 0.3) (Table 2). The sex of the person did not have any impact (female: seizure-alert, 52.9%; general population, 61.4%; χ^2^ = 0.513, *p* > 0.3). Neither age of the pet dog at arrival at the person’s home (e.g., 2 to 5 months: seizure-alert, 33.3%; general population, 51.6%), nor the provenance of the pet dog (such as breeder, rescued; e.g., breeder: seizure-alert, 33.3%; general population, 34.4%) appeared to have any effect (all chi-squared, *p* > 0.1). 

The 22 dogs reported as spontaneously alerting also exhibited response behaviors. During the seizure, most of them stayed close to their owner (50%), and this is also the most frequent behavior exhibited by seizure-response dogs (i.e., the only demonstrated specific behavior during or immediately after a seizure) (63.3%). While in both groups, dogs tended to bark (35% and 40%) and/or pace around (30% and 36.6%), seizure-alert dogs were described mostly as staring at their owner (40%) and going to another person (30%). On the other hand, seizure-response dogs licked their owner’s face (46.7%) and/or were anxious (43.3%).

### 3.4. Monash Canine Personality Questionnaire Refined

**Figure 2:**
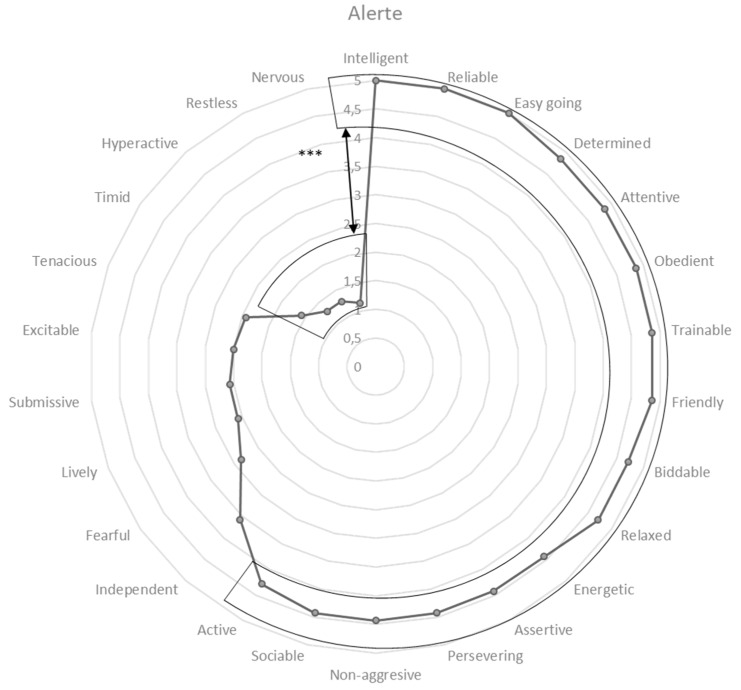
Behavioral profile of seizure-alert dogs. Score differences between behavioral items for seizure-alert dogs were assessed with a Kruskall–Wallis test, *** < 0.0001.

Data for 24 dogs were analyzed, including 7 SAD and 17 NAD. Seizure-alert pet dogs’ behavioral traits with the highest scores were “positive” traits. Indeed, pet owners considered them as smart, reliable and easy to live with (i.e., noted with a mean of 5 ± 0) as well as determined, attentive, obedient, trainable and friendly (i.e., noted with a mean of 4.85; for other, see Figure 2). On the contrary, SAD owners attributed low scores to the “negative” items, such as nervous (mean = 1.1 ± 0.37), restless (mean = 1.3 ± 0.48), hyperactive (mean = 1.3 ± 0.48), shy (mean = 1.6 ± 1.5), and stubborn (mean = 2.4 ± 0.97). The opposition between positive behaviors scores (e.g., intelligent, attentive; positive group in Figure 2) and negative behaviors scores (e.g., nervous, restless; negative group in Figure 2) was significant when comparing any of the variables of one group to any variable of the other (Kruskal Wallis test, *p* < 0.0001).

**Figure 3:**
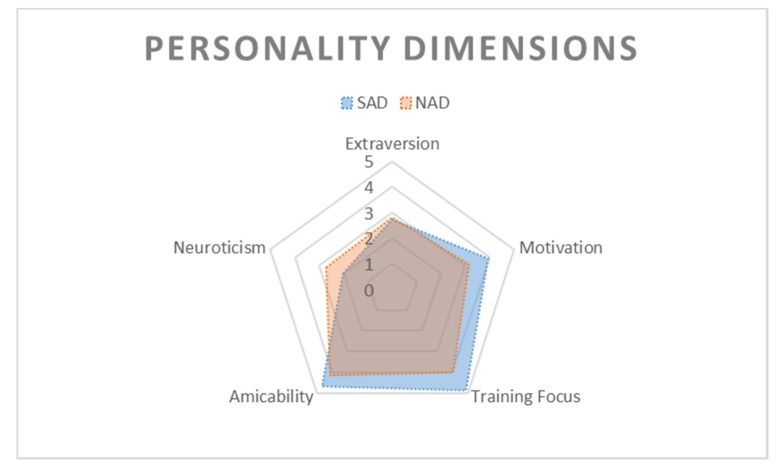
Personality dimensions: Representation of seizure-alert dogs (SAD) and non-alerting dogs (NAD) on the five dimensions of the Monash Canine Personality Questionnaire-Revised (MCPQ-R).

Significant differences were found between spontaneously alerting dogs and non-alerting dogs for the traits Motivation (n = 24, U_mean_ = 98, p_mean_ = 0.012), Training Focus (n = 24, U_mean_ = 100.5, p_mean_ = 0.007) and Neuroticism (n = 24, U_mean_ = 27, p_mean_ = 0.037). The scores for Motivation and Training Focus were significantly higher for SAD (Mdn_mean_ = 4, and Mdn_mean_ = 5, respectively) than for NAD (Mdn_mean_ = 3.2, and Mdn_mean_ = 4.2) whereas scores for Neuroticism were significantly lower for SAD (Mdn_mean_ = 1.75) than for NAD (Mdn_mean_ = 2.5). We found no significant differences for Extraversion (p_mean_ = 0.868) and Amicability (p_mean_ = 0.118). SAD and NAD are represented in Figure 3 on the basis of the five dimensions described in the MCPQ-R.

### 3.5. Monash Dog–Owner Relationship Scales

The total Dog–Owner Relationship score, calculated using the three subscales, was significantly higher for SAD (Mdn = 4.3) than for NADs (Mdn = 3.6) (U = 95, p = 0.024). Although there were no significant differences for the subscale “Interaction” (p_mean_ = 0.125), the score for the subscale “Perceived Costs” was significantly lower for SAD (Mdn_mean_ = 1.2) than for NAD (Mdn_mean_ = 1.7) (n = 24, U_mean_ = 28, p_mean_ = 0.043,). Finally, for the subscale “Emotional Closeness”, scores tended to be higher for SAD (Mdn_mean_ = 4.7) than for NAD (Mdn_mean_ = 4) (n = 24, U_mean_ = 90, p_mean_ = 0.054).

### 3.6. Toward Behavioral Profiles of Seizure-Alert

**Figure 4:**
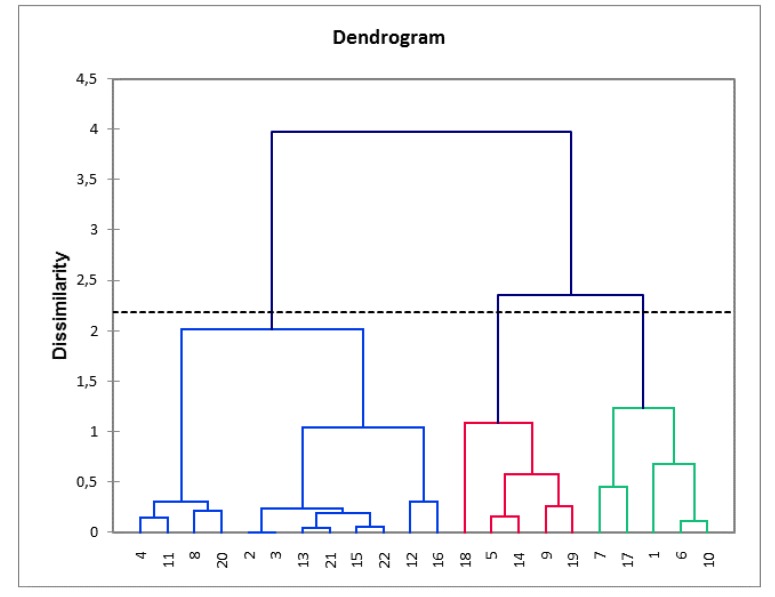
Dendrogram plot generated from the hierarchical cluster analysis of alert behaviors of the population study: Clusters distribution.

As mentioned above, spontaneous seizure-alert pet dogs can exhibit a variety of behaviors during an alert. We performed a multiple correspondence analysis to establish potential individual profiles. A dendrogram (Figure 4) visualizes the solution with the greatest distance between clusters. This resulted in a model with three clusters (groups). Group I accounted for 54.5% (n = 12) of the population and Group II and III for 22.7% (n = 5 for each) of the sample population (Figure 1).

**Figure 5:**
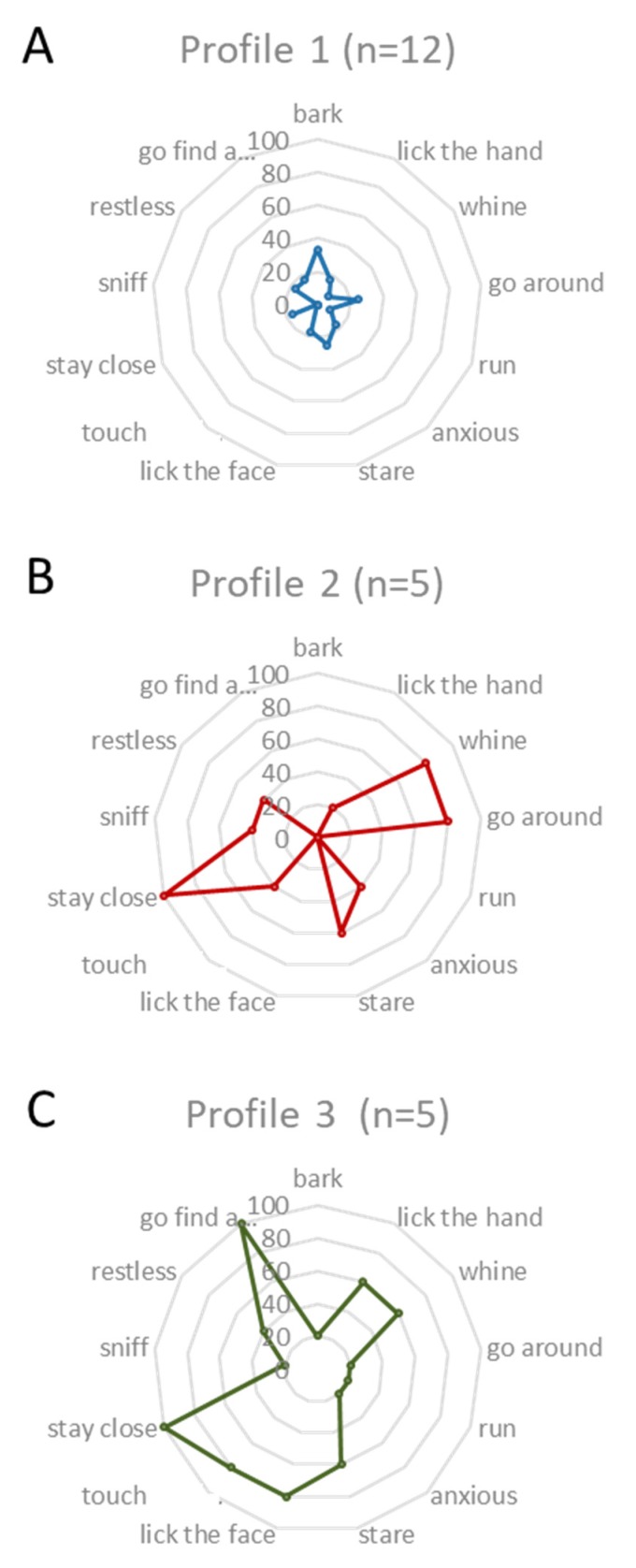
Behavioral profiles of seizure-alert pet dogs of (**A**) group 1 (**B**) group 2 and (**C**) group 3 obtained after a cluster analysis.

The behaviors performed by the different clusters were investigated (Figure 5) and compared based on other variables. No variable was found to have a statistical influence (participant gender, age of arrival of pet dog in the house, sex of the pet dog, time spent with the pet dog per day, neutering status, pure or mixed breed, size, provenance of the pet dog, being in charge of feeding, going out or training, type of epilepsy, experience of auras, feeling the seizure coming, frequency of seizures, time of the day when the seizure occurs, age of the owner, time of first alert, reliability in alerting, timing of the alert).

The first profile (n = 12; Figure 5A) included pet dogs who presented few seizure-alert behaviors, on average two behaviors (SD = 0.33). They mostly barked (33.3%), went around (25%) and stared at the person (25%). The pet dogs with this profile never demonstrated the behaviors “touch with the paw” or “sniff”.

Pet dogs with both the second and third profiles had maximum scores for “stay close” (100% each) but differed for other traits.

The second profile (n = 5; Figure 5B) included pet dogs that mainly whined and went around the person (80% each). Pet dogs with the second profile did not bark, run, lick face, or go to another person.

The third profile (n = 5; Figure 5C) included pet dogs that went towards a relative or caretaker (100%) but also displayed physical contact as lick face (80%) and touch with paw (80%). Pet dogs with this profile, at different rates, demonstrated all described behaviors.

## 4. Discussion

This study, based on self-report questionnaires by a population of French people with epilepsy, aimed to characterize spontaneously seizure alerting by pet dogs and to assess the replicability of the results of previous studies as this represents one of the most crucial challenges in science [24]. Across a series of questions asked to owners who reported having spontaneously seizure-alerting pet dogs, we established the profile of this type of pet dog (during and outside alerts) and the potential influence of owners’ dependent factors.

### 4.1. Owner-Related Factors

Interestingly, no owner-related factor related to SAD could be identified. This suggests that any pet dog of any person could develop alert behaviors. This may even explain the recent report that trained dogs discriminated the seizure odor perfectly despite the variety of seizures and of individual odors [25]. More precisely, our results showed that alerting behavior was unrelated to owner traits such as gender or age, as reported in other studies [11,15,26,27]. No differences could be evidenced concerning alert circumstances, although, for these results, it is not possible to rule out the effect of a limited sample size or that some alerts could go unnoticed by epileptic people in particular situations (e.g., night, physical barrier).

All main types of epileptic seizures were represented in our sample, with no significant variations whether the pet dog developed alert behaviors or not. This suggests that pet dogs seem able to alert all types of seizures. Dogs have been reported to alert spontaneously for each of the different kinds of seizures classified by the authors as “person is completely unconscious and falls down”, “person does not fall down but cannot respond normally to environment”, “person is able to respond normally to environment” and “seizure that no one else notices” [15]. Earlier studies reported that spontaneously seizure-alerting pet dogs as well as service seizure-alert pet dogs are able to alert tonic-clonic seizures and focal seizures with impaired awareness [11,27,28] or atonic and absence seizures [11]. All studies, including ours, converge, therefore, to show that dogs can develop alert behaviors for any type of seizure, a finding in accordance with the recent discovery that trained dogs can discriminate a seizure odor independently of the person and type of seizure [25]. Dalziel et al. [13] suggested that dogs were more likely to alert for focal seizures with impaired awareness seizures associated with migraines and/or auras/symptoms (patient-reported), such as an indescribable weird feeling in their head, nausea, lip smacking or mouth movements, and changes in breathing. This suggests the possibility that the dog might actually be reacting to the aura instead of alerting to a seizure [9], and would concord with Martos et al.’s report [15], showing that a large majority of “preictal” symptoms occurred less than one minute (47% for SAD owners, 41% for NAD owners) or between one to five minutes (21% for SAD owners and 28% for NAD owners) before a seizure while there was a linear association between time between preictal symptoms and seizure and times between alerting behaviors and seizures. Nevertheless, 22% of spontaneously alerting dogs’ anticipation time was superior to 30 min before the seizure, and it represented 63% of trained dogs; the other 37% alerting from 10 min to 30 min before the ictus event.

Altogether, these results suggest that although a majority of dogs improperly categorized SAD are in fact probably reacting to auras (i.e., a focal seizure involving subjective sensory or psychic phenomena only [18], usually short, lasting for a few seconds to a few minutes). Some untrained dogs and a majority of trained dogs were able to predict a seizure, presenting a specific behavior outside the ictus state, maybe coinciding with a prodromal state. A prodrome lasts from hours to days and is defined as a pre-ictal phenomenon, a subjective or objective clinical alteration that heralds the onset of an epileptic seizure but does not form part of it [29,30]. In our study, auras (as well as feeling a seizure coming) were as frequent in both the overall population and the seizure-alerting pet dog owners.

### 4.2. Pet Related Factors

We found no significant differences in pet dogs’ characteristics (e.g., breed, sex, reproductive state, age, origin) between alerting pet dogs and the overall population. Here again, our results are consistent with those of previous studies [11,13,15].

The MCPQ-R revealed that personality traits differed between SAD and NAD. As in Martinez-Caja et al.’s study [15], SAD’s scores were higher for Motivation and Training Focus, and tended to be higher for Amicability than NAD’s scores. Their scores were lower for Neuroticism. As mentioned in the Introduction, some trainers or charities training SAD said they looked for dogs described as “sensitive” with some anxious traits. Our findings suggest that these dogs would not be the best suited for developing spontaneous alert behaviors as the dogs that developed alert behaviors had lower scores for Neuroticism. In addition, selecting anxious dogs raises ethical concerns as they can represent a danger for both themselves and the human with epilepsy. Indeed, it seems logical to think that such a dog would react adversely to human seizures with negative consequences for the dyad (e.g., death of the dog from an overwhelming stress reaction, aggressive behaviors toward humans) [31].

Our findings converge with studies on behavioral selection of service dogs. For example, Duffy and Serpell [32] found that dogs prone to excitability, stranger-and dog-directed aggression, and social and nonsocial fear were less likely to complete training successfully. A review [33] suggested that boldness is an important trait for successful completion of working tasks by dogs working as scent detection dogs for conservation, individuals with low boldness score being more fearful, anxious and easily distracted, although the literature is too scarce to be able to draw any definite conclusions.

Pet dogs here presented three types of behavioral profiles when alerting, some pet dogs were presented only one to two behaviors, and pet dogs, with the two other profiles presented more behaviors. Important inter-individual variability of behaviors during alert had been mentioned previously [11,13,14], but here for the first time, we describe behavioral profiles, grouping individuals with similar behaviors. It would be interesting now to investigate, on a larger sample size, the relationships between these profiles and personality traits that could influence the type of reaction and more largely, the way a pet dog perceives a seizure of its owner. We hypothesize, for example, that profile B dogs (i.e., dogs that stayed close, whined and went around the person) would have slightly lower scores for traits related to self-confidence. The influence of differences in type of relationship of the dyads also requires investigation. Although our results are only indicative and our sample was small, this particular point of profile groups, requires further examination.

### 4.3. Human–Dog Relationships

The human–dog bond evidenced in this study confirmed the results of a previous study [15], as the Total MDORS score was significantly higher for SAD owners. This suggests that the quality of dog ownership is influenced by the owner’s perception of the alerts given by the dog, and it also influenced the perception of the costs, as this “Perceived Costs” subscale varied according to the presence or absence of alerting behaviors. However, contrary to this previous study [15], we found no differences concerning the extent to which owners and pet dogs engaged in shared activities (“Dog–Owner Interaction” subscale). However, our data indicated that the perceived emotional closeness of the relationship (“Perceived Emotional Closeness” subscale) was significantly impacted by the presence or absence of alerting behaviors. This subscale included items relating to social support, affectional bonding, psychological attachment and companionship [16]. Interestingly, various studies (for a review see [25]) reported that willingness or seeking to bond were crucial factors in training centers’ or charities’ choice of both future service dogs and future owners. It would be interesting to explore this feature, applying experimental tests, for example, in order to discard the perceptual aspect as well as the owners’ view and analyze bonding behavior directly.

### 4.4. Limitations

One important challenge in studies based on a questionnaire survey and, in addition, on such a controversial (i.e., SAD) and complex (i.e., participants with epilepsy) topic, is to evaluate how subjective responses are. For example, we cannot be sure that the alerts are efficient, that behaviors are reported correctly, or that we have a proof that pet dogs really did alert a seizure. Another limitation that must be considered when interpreting the data is that they were based only on anonymous self-reporting; allowing no possible verification of medical aspects of epilepsy (e.g., diagnosis, seizure types). Self-reporting is a question that has already been mentioned as an issue with questionnaires on this subject [34]. Nevertheless, our results were consistent with earlier studies, suggesting that general traits may have been found.

### 4.5. Future Research Directions

For 20% to 30% of people with epilepsy, seizures are either intractable or uncontrolled or they suffer from significant adverse side effects to medication [35]. In addition, various comorbid psychiatric disorders are commonly associated with epilepsy, such as anxiety and depression [36]. Therefore, research on seizure-alert dogs could have an important impact on the life of thousands of people as their presence can at least increase the quality of their lives [11,13,28,37] and maybe thus even reduce the frequency of seizures [27,28,37]. However, much research is still needed, as many questions still remain unanswered, such as the efficiency of an alert (e.g., specificity of alert behaviors and reliability), or the specificity of the outcomes, such as improvement in quality of life. More precisely, characterization of the dogs appropriate for such work is still needed.

In particular, future research could use cognitive measures to assess the profiles of these dogs, as their abilities developed spontaneously without training, which is probably demanding in terms of cognitive flexibility. For example, using odor discrimination, the detour and pointing tasks, or even the unsolvable task, has proved interesting for the selection of some service dogs [38].

## 5. Conclusions

This study highlighted the fact that, using a specific questionnaire or standardized questionnaires, no particular differences could be found concerning the characteristics of dogs (e.g., sex, breed), owners (e.g., sex, age) or epilepsy (e.g., seizure frequency) between dogs that developed alert behaviors spontaneously and those that did not. Nevertheless, SAD’s scores were higher for “Motivation” and “Training Focus” and lower for “Neuroticism” in the personality questionnaire. In addition, owners of SAD scored their human–dog bond higher than owners of non-alerting dogs. Our results confirm those obtained by previous authors for other populations, their robustness, and expanded the question of alert behaviors, behavioral profiles and circumstances in which the behaviors could occur.

Together with the recent discovery of dogs’ abilities to discriminate a seizure odor, they open further lines of research on the selection and training of service dogs dedicated to seizure-alerting.

## Figures and Tables

**Table 1:** Demographics of Dog Owners in our study sample.

Dog Owners	N Alert (n = 17)	N Total (n = 57)	N without Answer (n = 15)	% of the Study Population
**SEX**				
male	8	22		38.6
female	9	35		61.4
**Mean Age Mean 29.25 ± 13.46 Years Old**				
<25 years	7	25		43.9
25 to 40 years old	6	18		31.6
41 to 65 years old	4	13		22.8
>65 years old	0	1		1.8

**Table 2:** Demographics of epilepsy in our study sample and specifically in the human population who reported alert behavior of their dog.

Seizure Duration	N Alert	% Alert	N	% of the Study Population	N without Answer
	n = 16		n = 52		n = 20
1 s–1 min	1	6.25	12	23.08	
1–2min	4	25	12	23.08	
2–3 min	1	6.25	3	5.77	
3–4 min	0	0	1	1.92	
4–5 min	6	37.5	14	26.92	
5–10min	3	18.75	7	13.46	
>10	1	6.25	3	5.77	
**Frequency**	**n = 17**		**n = 58**		**n = 14**
Per day	4	23.53	18	31.03	
Per week	4	23.53	11	18.97	
Per month	5	29.41	20	34.48	
Per year or none	4	23.53	9	15.52	
**Trigger %**	**n = 21**		**n = 46**		**n = 26**
Light	6	28.57	5	10.87	
Noise	3	14.29	2	4.35	
Heat	4	19.05	10	21.74	
**Time %**	**n = 21**		**n = 46**		**n = 26**
Wake up	6	28.57	9	19.56	
Morning	6	28.57	8	17.39	
Afternoon	3	14.29	5	10.86	
Day	6	28.57	14	30.43	
Night	5	23.81	8	17.39	
Sleep	7	33.33	11	23.91	
**Auras**	**n = 16**		**n = 54**		**n = 18**
Yes	9	56.25	27	50.00	
No	5	31.25	23	42.59	
Don’t know	2	12.5	4	7.41	
**Feel the Seizure is Coming**	**n = 17**		**n = 55**		**n = 17**
Yes	8	47.06	28	50.91	
No	8	47.06	25	45.45	
Don’t know	1	5.88	2	3.64	
**Seizure Frequency**	**n = 17**		**n = 58**		**n = 14**
Daily	4	23.53	18	31.03	
Weekly	4	23.53	11	18.97	
Monthly	5	29.41	20	34.48	
Yearly	4	23.53	9	15.52	
**Seizure Type**	**n = 16**		**n = 35**		**n = 37**
Generalized onset	12	75	20	57.14	
Focal aware seizures	1	6.25	3	8.57	
Focal seizures with impaired awareness	1	6.25	1	2.86	
Multiples	2	12.5	11	31.43	

**Table 3:** Demographics of dogs in our study sample.

Dogs	N Alert	% Alert	N Total	% of the Study Population	N without Answer
**SEX**	**n = 20**		**n = 68**		**n = 4**
Male	6	30	21	30.88	
Female	3	15	12	17.64	
M neutered	5	25	16	23.52	
F neutered	6	30	19	27.94	
Total male	11	55	37	54.41	
Total female	9	45	31	45.59	
**Breed**	**n = 19**		**n = 65**		**n = 7**
Cross bred	5	26.32	17	26.15	
Pure bred	14	73.68	48	73.85	
**SIZE**	**n = 19**		**n = 60**		**n = 12**
<5 kg	2	10.53	8	13.33	
5–9 kg	2	10.53	10	16.66	
10–19 kg	5	26.32	13	21.66	
20–29 kg	3	15.79	10	16.66	
30–39 kg	5	26.32	9	15	
40–49kg	0	0.00	4	6.66	
50 to >50 kg	2	10.53	6	10	
**Average of Time Spent Close**	**n = 20**		**n = 64**		**n = 8**
0–5 h	3	15	15	23.44	
6–11 h	6	30	15	23.44	
12–17 h	3	15	14	21.87	
18–24 h	8	40	20	31.25	
**Are You in Charge (% of yes)**					
feeding		68.18		54.2	
going out		63.64		56.9	
training		54.55		40.3	
**Age of Arrival**	**n = 18**		**n = 60**		**n = 12**
< 2 months	2	11.11	6	10.00	
2–5 months	6	33.33	31	51.67	
6 months to 1 year	4	22.22	8	13.33	
> 1 year	6	33.33	15	25.00	
