# Erratum: Catala, A., et al. Is There a Profile of Spontaneous Seizure-Alert Pet Dogs? A Survey of French People with Epilepsy. *Animals* 2020, *10*, 254

**DOI:** 10.3390/ani11020340

**Published:** 2021-01-29

**Authors:** Amélie Catala, Patrick Latour, Ana Martos Martinez-Caja, Hugo Cousillas, Martine Hausberger, Marine Grandgeorge

**Affiliations:** 1 CNRS, EthoS (Éthologie Animale et Humaine)-UMR 6552, University Rennes, Normandie University, F-35000 Rennes, France; hugo.cousillas@univ-rennes1.fr (H.C.); martine.hausberger@univ-rennes1.fr (M.H.); marine.grandgeorge@univ-rennes1.fr (M.G.); 2 Association Handi’Chiens, 13 Rue de l’Abbé Groult, 75015 Paris, France; 3 Etablissement Médical de la Teppe, 25 Avenue de la Bouterne, 26600 Tain-l’Hermitage, France; patrick.latour@teppe.org; 4 Department Nutrition, Genetics and Ethology, Faculty of Veterinary Medicine, Ghent University, Heidestraat 19, B-9820 Merelbeke, Belgium; ana.martos@ugent.be

The authors wish to make the following corrections to this paper:

## 1. The Authorship

Amélie Catala ^1,2,^*, Patrick Latour ^3^, Ana Martos Martinez-Caja ^4^, Hugo Cousillas ^1^, Martine Hausberger ^1^ and Marine Grandgeorge ^1^

1 CNRS, EthoS (Éthologie Animale et Humaine)-UMR 6552, University Rennes, Normandie University, F-35000 Rennes, France

2 Association Handi’Chiens, 13 Rue de l’Abbé Groult, Paris, France

3 Etablissement Médical de la Teppe, 25 Avenue de la Bouterne, 26600 Tain-l’Hermitage, France

4 Department Nutrition, Genetics and Ethology, Faculty of Veterinary Medicine, Ghent University, Heidestraat 19, B-9820 Merelbeke, Belgium

## 2. The Email of Corresponding Author

Amélie Catala: amelie.catala@gmail.com

## 3. Author Contributions

Conceptualization, A.C., H.C., M.H. and M.G.; methodology, A.C., H.C., A.M.M.-C., M.H. and M.G.; software analysis, A.C.; validation A.C., H.C., M.H. and M.G.; formal analysis, A.C., H.C., M.H. and M.G.; investigation, A.C., P.L.; resources management, A.C., P.L.; data curation, A.C.; writing—original draft preparation, A.C.; writing—review and editing, A.C., H.C., M.H., M.G., P.L.; visualization, A.C.; supervision, A.C., H.C., M.H., M.G., P.L.; project administration, A.C., H.C., M.H., P.L., M.G. All authors have read and agreed to the published version of the manuscript.
